# A Case of Disseminated Nocardiosis due to *Nocardia otitidiscaviarum* With Pulmonary Infection and a Thyroid Abscess

**DOI:** 10.1002/rcr2.70663

**Published:** 2026-06-25

**Authors:** Seigo Miyoshi, Miyuki Tanabe, Tetsuya Yamamoto, Mayuko Semba, Chika Sato, Akira Watanabe, Sohei Kitazawa, Ryoji Ito

**Affiliations:** ^1^ Department of Respiratory Medicine National Hospital Organization Ehime Medical Center Ehime Japan; ^2^ Department of Molecular Pathology Ehime University Graduate School of Medicine Ehime Japan

**Keywords:** disseminated nocardiosis, *Nocardia otitidiscaviarum*, thyroid infection

## Abstract

A 64‐year‐old man without relevant medical history was admitted with appetite loss and dyspnea. Chest CT showed a 5 cm right upper lobe mass and diffuse granular shadows throughout the lungs. Initial treatment targeted suspected pneumonia with lung cancer, but cultures from admission blood and sputum samples revealed *Nocardia* on day three, leading to oral trimethoprim‐sulfamethoxazole. Despite therapy, his respiratory condition worsened, and he died on the 10th day of hospitalization. Autopsy demonstrated squamous cell carcinoma in the right upper lobe. The diffuse pulmonary nodules showed neutrophil infiltration and focal necrosis, consistent with necrotizing pneumonia, and similar lesions were found in the thyroid. The cultured organism was later identified as *Nocardia otitidiscaviarum* , and the cause of death was determined to be disseminated nocardiosis. *Nocardia* infection is an infrequent disease that can have a fatal course; therefore, clinicians must consider the diagnosis early, perform rapid bacteriological testing and initiate timely and appropriate treatment.

## Graphical abstract

*Nocardia otitidiscaviarum* is a rare pathogen worldwide compared with other *Nocardia* species. Although *Nocardia* infections occur mainly in immunocompromised individuals and typically involve lungs and skin, cases in immunocompetent hosts have also been reported in recent years. Thyroid infection is particularly uncommon due to the gland's inherent resistance, but this report describes a disseminated *N. otitidiscaviarum* infection with pulmonary involvement and a thyroid abscess.

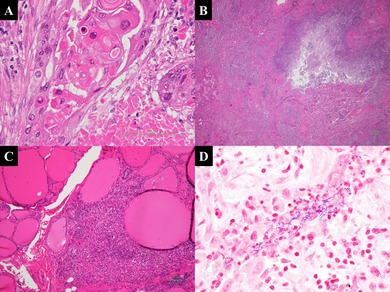

## 1 Introduction

Nocardiosis is caused by a group of opportunistic bacterial pathogens belonging to the genus *Nocardia*. These pathogens are slow‐growing, gram‐positive and partially acid‐fast, with filamentous branching and environmental ubiquity [1]. Compared with other *Nocardia* species, *Nocardia otitidiscaviarum* ( *N. otitidiscaviarum* ) is a rare pathogen worldwide [1, 2, 3].

*Nocardia* infection has been reported mainly in immunocompromised individuals, but infection in immunocompetent individuals has also been reported in recent years [1, 4, 5]. The species commonly affect the lungs and skin [1, 6]. Thyroid infections are rare because the thyroid gland is a resistant organ owing to its encapsulated position, high blood flow, and the bactericidal action of iodine and hydrogen peroxide [6]. Here, we describe a case of disseminated *N. otitidiscaviarum* infection with pulmonary infection and a thyroid abscess.

## 2 Case Report

**FIGURE 1:**
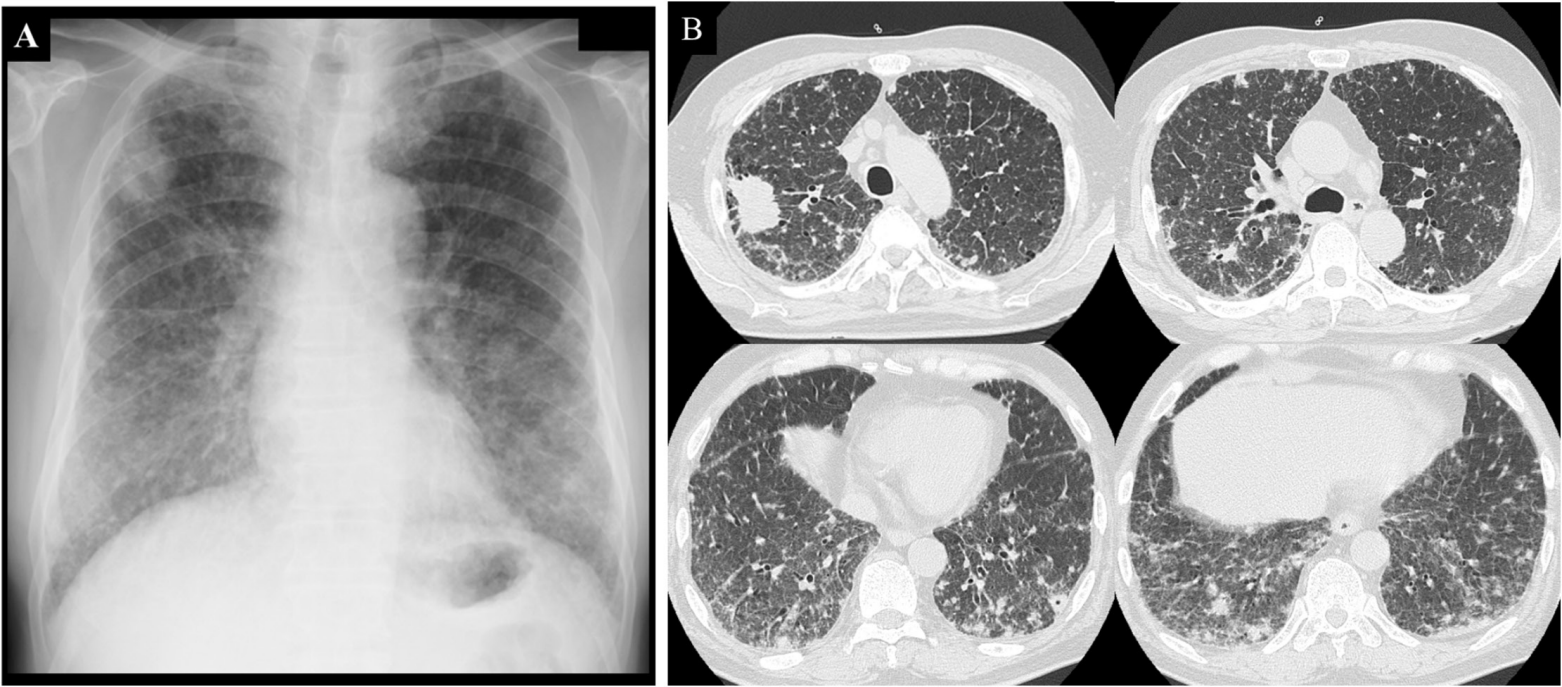
(A) Chest radiography performed on admission showed a mass shadow in the right upper lung field and small granular shadows in the bilateral lung fields. (B) Chest computed tomography also showed random distribution of granular shadows and nodules of 1–12 mm in size in both lungs, and nodule shadows with cavities in some of them. In addition, some infiltration shadows were shown under the pleura of both lower lobes. A 5‐cm large marginal irregular mass shadow was also observed in the upper right lobe.

**FIGURE 2:**
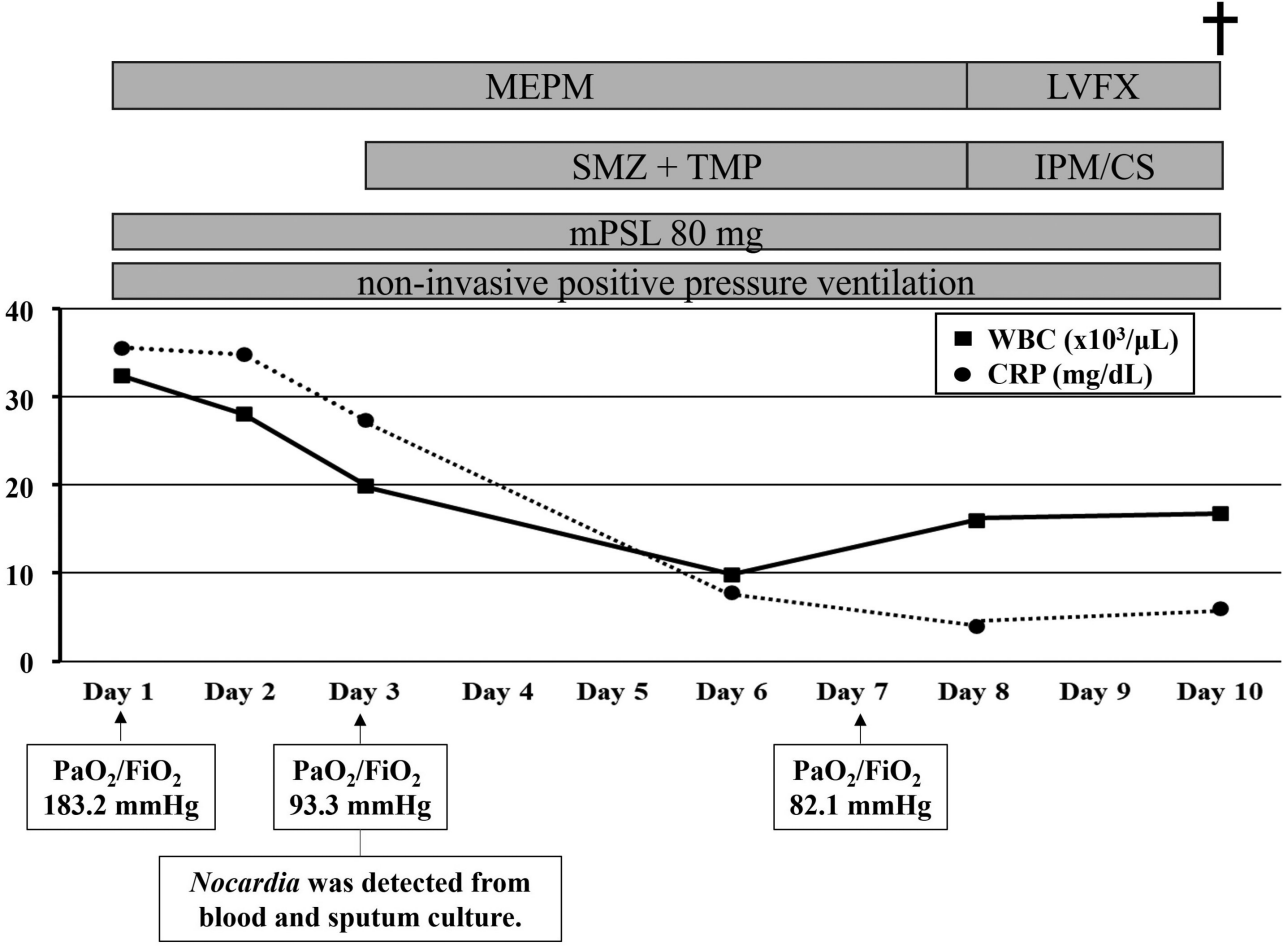
Clinical course after hospitalization. CRP, C‐reactive protein; IPM/CS, imipenem‐cilastatin; LVFX, levofloxacin; MEPM, meropenem; mPSL, methylprednisolone; SMZ, sulfamethoxazole; TMP, trimethoprim; WBC, white blood cell.

A 64‐year‐old male smoker with no relevant medical history presented with a one‐month history of anorexia and dyspnea. On admission, he was mildly febrile (37.5°C) and hypoxic (SpO_2_ 90% on 2 L/min O_2_). There were no clear physical findings, such as thyroid enlargement. No neurological symptoms or signs were observed. Laboratory tests showed marked leukocytosis (32.4 × 10^3^/μL), significantly elevated C‐reactive protein (35.56 mg/dL), mild liver dysfunction, and reduced renal function (eGFR 46.9 mL/min/1.73m^2^). Mild diabetes mellitus was observed (HbA1c 6.7%). Both the HIV antigen and antibody tests were negative. Chest X‐ray revealed a 5‐cm mass shadow in the right upper lobe and diffuse granular shadows throughout both lungs (Figure 1A). Chest CT findings showed random distribution of granular shadows and nodules measuring 1–12 mm in size in both lungs, and nodule shadows with cavities in some of them (Figure 1B). In addition, subpleural infiltrative shadows were observed in both lower lobes. A 5‐cm irregularly marginated mass was also observed in the upper right lobe. Based on these findings, a combination of lung cancer, pulmonary metastases and pneumonia were suspected. There were no findings suggestive of a thyroid abscess. Empiric antibiotics were started. However, his respiratory condition worsened on the night of admission, and NIPPV and corticosteroids were subsequently initiated (Figure 2).

On hospital Day 3, blood and sputum cultures collected at admission grew *Nocardia* spp., prompting initiation of oral trimethoprim‐sulfamethoxazole (TMP‐SMX). Although inflammatory markers decreased, his respiratory failure progressed. By Day 7, he became unable to take oral medications and exhibited progressive renal dysfunction (eGFR decreased from 46.9 to 30.7 mL/min/1.73m^2^); TMP‐SMX and MEPM were discontinued and intravenous imipenem‐cilastatin and levofloxacin were initiated. These agents have been reported as recommended alternative therapies when TMP‐SMX cannot be continued in the treatment of *Nocardia* infection [4]. Renal dysfunction and thrombocytopenia precluded the use of amikacin or linezolid. Despite therapy, respiratory status worsened, and he died on Day 10.

**FIGURE 3:**
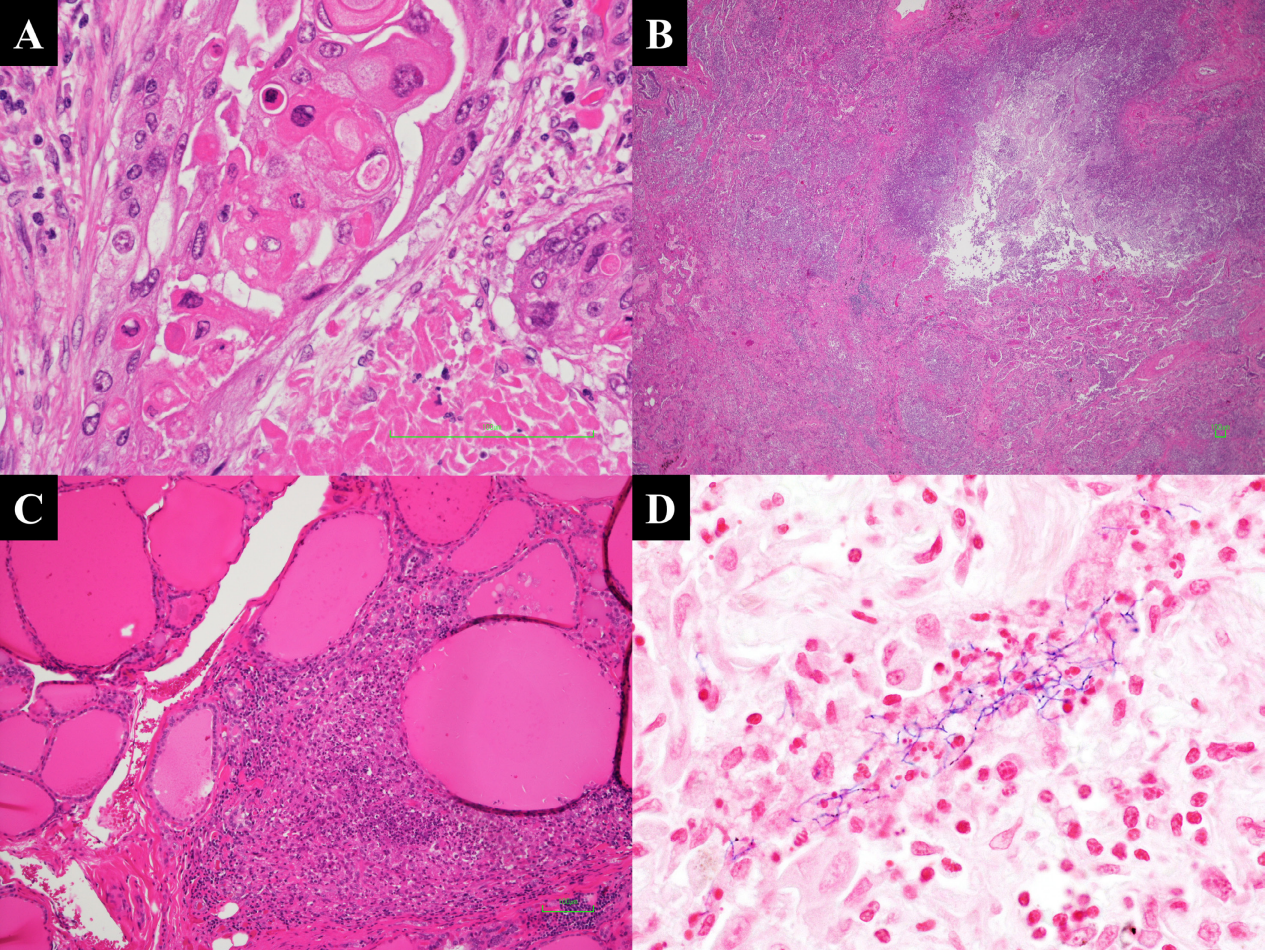
(A) A postmortem microscopic examination showed large cells with keratinization in the mass in the right upper lung lobe, which was diagnosed as squamous cell carcinoma (haematoxylin and eosin [HE] staining, 400 × magnification). (B) The granular nodules found throughout the lungs were accompanied by neutrophil infiltration and necrosis in some parts, which indicated necrotizing pneumonia (HE, 40 × magnification). (C) The accumulation of neutrophils and necrotic substances were also noted in the thyroid gland, which led to the diagnosis of a thyroid abscess (HE, 100 × magnification). (D) Gram staining of the lung tissue showed gram‐positive filamentous bacteria (Gram stain, 400 × magnification).

Postmortem macroscopic examination revealed a hard white mass in the right upper lung and granular nodules throughout both lungs. Microscopic examination revealed large, keratinized cells in the mass in the right upper lobe, which was diagnosed as squamous cell carcinoma (Figure 3A). The granular nodules found in the lungs were accompanied by neutrophil infiltration and necrosis consistent with necrotizing pneumonia (Figure 3B). Similar findings were observed in the thyroid gland (Figure 3C). Gram staining of the lung tissue (Figure 3D) and thyroid gland tissue showed gram‐positive filamentous bacteria. The *Nocardia* species detected in the sputum and blood cultures was found to be *N. otitidiscaviarum* after the patient's death.

The final diagnosis was disseminated nocardiosis involving the lungs and thyroid, complicated by underlying lung squamous cell carcinoma. The patient's death was attributed to progressive disseminated nocardiosis despite antimicrobial therapy.

## 3 Discussion

This report describes a fatal case of disseminated *N. otitidiscaviarum* infection in a patient who also had primary lung cancer. *Nocardia* species are gram‐positive, partially acid‐fast, filamentous bacteria [1] commonly found in the soil, vegetation and organic matter. Although human nocardiosis is relatively rare, it can cause severe and life‐threatening disease, particularly in immunocompromised individuals [1, 2]. The prevalence of specific *Nocardia* species varies geographically, and infections caused by *N. otitidiscaviarum* remain rare in many areas [1, 2, 3]. In Japan, *N. otitidiscaviarum* accounts for only 7% of all nocardiosis cases [3].

To date, there have been only 12 reports of thyroid nocardiosis [6, 7]. The most common infection site of nocardiosis is the pulmonary system or skin, because it typically develops owing to the inhalation of mycelial fragments or exposure to the causative bacteria due to cuts or abrasions, eventually resulting in other organs dissemination [1, 6]. Thyroid infection is unusual due to several protective features: the gland is encapsulated, has a rich blood supply, and contains iodine and hydrogen peroxide, both of which have bactericidal effects [6]. Previously reported cases of thyroid nocardiosis predominantly occurred in patients with significant immunosuppression, including those with long‐term corticosteroid use, diabetes mellitus, or after organ transplantation [6, 7]. The present case was also complicated with diabetes mellitus and lung cancer in the upper right lobe. Nevertheless, cases of nocardiosis have also been reported in immunocompetent individuals [1, 4, 5], clinicians should remain vigilant caution regardless of the patient's immune status.

It has been reported that chest CT findings of pulmonary nocardiosis often include infiltrative shadows, nodules, masses and cavitation [8]. These findings require differentiation between vasculitis, malignant diseases, and other infectious diseases including tuberculosis and mycosis [8]. In addition, the presence of cavitation and pleural effusion has been reported to differ significantly between localized and disseminated pulmonary nocardiosis [9]. In this case, metastatic lung tumours caused by primary lung cancer were also considered as differential diagnoses, as mass shadows were observed in the upper right lobe and random granular shadows, nodules and cavitation were found in both lungs.

Management of nocardiosis typically requires prolonged antimicrobial therapy, often using sulfonamide‐based regimens for 6–12 months [1, 2]. However, antimicrobial susceptibility varies widely among *Nocardia* species [1, 2, 3]. The susceptibility of *N. otitidiscaviarum* to TMP‐SMX varies from 50% to 87% [2]. The species is typically sensitive to amikacin and linezolid while demonstrating high resistance to imipenem, ceftriaxone, and ciprofloxacin [2]. The strain in this case also showed resistance to many antibiotics, except gentamicin and minomycin (Table 1). No susceptibility test for TMP‐SMX was performed; therefore, the susceptibility status of the isolate is unknown.

**TABLE 1:** Antimicrobial susceptibility test result in the present case.

Antibiotic	MIC (μg/mL)	Interpretation
penicilin	> 4	Resistance
sulbactam‐ampicillin	> 16	Resistance
imipenem	> 8	Resistance
cefazolin	> 16	Resistance
cefotiam	> 16	Resistance
cefotaxime	> 16	Resistance
gentamicin	≤ 1	Sensitive
erythromycin	> 4	Resistance
clindamycin	> 2	Resistance
minomycin	≤ 1	Sensitive
levofloxacin	> 4	Resistance

Early diagnosis and selection of appropriate antibiotics are critical, yet challenging. Conventional methods of species identification of *Nocardia* are microbiological examination of smeared specimens and culture tests. However, since *Nocardia* generally grows slowly, it takes 2–7 days to confirm colonies, and the final identification of the species may take about 4 weeks [5]. In this case, the identification of bacterial species and the results of antimicrobial susceptibility were unavailable before death, highlighting the diagnostic challenges associated with nocardiosis (In this case, *Nocardia* species identification was performed using biochemical testing, and ~1 month was required to obtain the results). Newer diagnostic methods, such as matrix‐assisted laser desorption ionization‐time‐of‐flight mass spectrometry (MALDI‐TOF MS) and next‐generation sequencing, may facilitate more rapid species identification. These techniques can identify bacterial species within minutes to hours [5, 10]. Additionally, the accuracy of these tests is high, reportedly close to 100% [3, 11, 12]. However, it has been reported that for less common *Nocardia* species, accuracy can be reduced in the MALDI‐TOF MS method, so caution is needed [12].

In conclusion, this case underscores the potential for severe disseminated *N. otitidiscaviarum* infection, even in apparently immunocompetent individuals, and highlights the importance of rapid diagnosis and tailored antimicrobial therapy. Additional case reports are needed to refine empirical treatment strategies, particularly for drug‐resistant strains.

## Author Contributions

All authors meet the ICMJE authorship criteria. S.M. contributed to conceptualizing the case report and drafted the manuscript. M.T., T.Y., M.S., C.S., and A.W. contributed data collection and revised the manuscript. S.K. contributed to the pathological assessment and revised the manuscript. R.I. contributed to supervising the study and revised the manuscript. All authors approved the final manuscript as submitted and agree to be accountable for all aspects of the work.

## Funding

The authors have nothing to report.

## Consent

The authors declare that written informed consent was obtained from the patient's family for the publication of this manuscript and accompanying images and attest that the form used to obtain consent from the patient(s) complies with the Journal requirements as outlined in the author guidelines.

## Conflicts of Interest

The authors declare no conflicts of interest.

## Data Availability

The data that support the findings of this study are available from the corresponding author upon reasonable request.
